# CpG Island Definition and Methylation Mapping of the T2T-YAO Genome

**DOI:** 10.1093/gpbjnl/qzae009

**Published:** 2024-02-01

**Authors:** Ming Xiao, Rui Wei, Jun Yu, Chujie Gao, Fengyi Yang, Le Zhang

**Affiliations:** College of Computer Science, Sichuan University, Chengdu 610065, China; College of Computer Science, Sichuan University, Chengdu 610065, China; Key Laboratory of Systems Health Science of Zhejiang Province, School of Life Science, Hangzhou Institute for Advanced Study, University of Chinese Academy of Sciences, Hangzhou 310024, China; CAS Key Laboratory of Genome Sciences and Information, Beijing Institute of Genomics, Chinese Academy of Sciences and China National Center for Bioinformation, Beijing 100101, China; University of Chinese Academy of Sciences, Beijing 100049, China; College of Computer Science, Sichuan University, Chengdu 610065, China; College of Computer Science, Sichuan University, Chengdu 610065, China; College of Computer Science, Sichuan University, Chengdu 610065, China; Key Laboratory of Systems Health Science of Zhejiang Province, School of Life Science, Hangzhou Institute for Advanced Study, University of Chinese Academy of Sciences, Hangzhou 310024, China

**Keywords:** T2T-YAO, Position-defined CpG island, Density-defined CpG island, DNA methylation, Genome analysis

## Abstract

Precisely defining and mapping all cytosine (C) positions and their clusters, known as CpG islands (CGIs), as well as their methylation status, are pivotal for genome-wide epigenetic studies, especially when population-centric reference genomes are ready for timely application. Here, we first align the two high-quality reference genomes, T2T-YAO and T2T-CHM13, from different ethnic backgrounds in a base-by-base fashion and compute their genome-wide density-defined and position-defined CGIs. Second, by mapping some representative genome-wide methylation data from selected organs onto the two genomes, we find that there are about 4.7%–5.8% sequence divergency of variable categories depending on quality cutoffs. Genes among the divergent sequences are mostly associated with neurological functions. Moreover, CGIs associated with the divergent sequences are significantly different with respect to CpG density and observed CpG/expected CpG (O/E) ratio between the two genomes. Finally, we find that the T2T-YAO genome not only has a greater CpG coverage than that of the T2T-CHM13 genome when whole-genome bisulfite sequencing (WGBS) data from the European and American populations are mapped to each reference, but also shows more hyper-methylated CpG sites as compared to the T2T-CHM13 genome. Our study suggests that future genome-wide epigenetic studies of the Chinese populations rely on both acquisition of high-quality methylation data and subsequent precision CGI mapping based on the Chinese T2T reference.

## Introduction

Position-sensitive and co-methylated CpG islands (CGIs) represent one of the several key epigenetic mechanisms for chromosome integrity and gene expression regulation [1,2]. To define highly variable CGIs based on both position and density genome-wide and to map their methylation status under different physiological conditions and in different cell types are prerequisite in most epigenomic studies [3–7]. Human reference genomes, especially those tailored to specific populations, provide single-base-resolution coordinates for genome-wide genetic and epigenetic mapping with ultimate precision [8]. The release of the telomere-to-telomere CHM13 (T2T-CHM13) genome in 2022 has provided such a benchmark by filling-up sequence gaps that contribute nearly 8% increase of the previous human genome assembly [9]. Therefore, it is of essence to compare it to the more recent human genome assembly of the Chinese population, the T2T-YAO genome [10], and to annotate all CGIs for both assemblies. Here, we simply raise two basic questions. First, are there significant differences in CGIs and their methylation between the two reference genomes? And if so, what are the relevant genes and their functions diverged between them? Second, does the T2T-YAO genome provide additional information or serve more appropriately as one of the reference genomes for the Chinese population? Both are of importance for subsequent genome-wide epigenetic studies and building methylation databases specifically for the Chinese populations under complex physiological and pathological conditions. We, therefore, chose to accurately predict genome-wide CGIs for both reference genomes and validate their applications in methylation studies by testing them on a limited number of datasets due to data availability and quality.

A genome-wide comparison of two reference genomes has been done recently between the classical GRCh38 and T2T-CHM13 genomes, and the authors discovered nearly 200 million base pairs unique to the T2T-CHM13 genome [9]. This result supports our effort in mapping the T2T-YAO and T2T-CHM13 genomes to explore their divergent sequences, and determining whether the divergent sequences are associated with specific biological functions becomes our first scientific question. We have described previously a genome-wide inclusive CGI definition, *i.e.*, a distance-based clustering method which yields short CpG clusters, known as position-defined CGIs showing potential gene regulatory functions and closely correlated with gene expression specificity [2,11,12]. Therefore, whether there is any specificity between T2T-YAO and T2T-CHM13 with respect to CGI features, becomes our second scientific question. It becomes feasible now to carry out a comparative analysis on the T2T-YAO and T2T-CHM13 genomes on CGI methylation profiles using public whole-genome bisulfite sequencing (WGBS) data [2,8,11,12], and this is what we address as the third scientific question.

To this end, we align the two genomes, compute both density-defined and position-defined CGIs, and map some genome-wide methylation WGBS data from representative organs to analyze differentially methylated CGIs between the two genomes. Our major findings are as follows: (1) there is an ∼ 4.7%–5.8% difference between the two genomes, which is composed of divergent sequence-associated genes correlated closely with neurological functions based on GO enrichment analysis [13–18]; (2) CGIs associated with the divergent sequences are significantly different with respect to CpG density and observed CpG/expected CpG (O/E) ratio [11,19]; (3) not only do WGBS data mapped to the T2T-YAO genome have a greater CpG coverage than those of the T2T-CHM13 genome, but also the T2T-YAO genome shows more hyper-methylated CpG sites than T2T-CHM13 does. Our study demonstrates statistically significant differences between T2T-YAO and T2T-CHM13 in genome sequences and epigenetic landmarks [20–22], such as CGI and methylation patterns, suggesting that the establishment of CGI and methylation profiles based on the Chinese T2T reference genome is crucial for subsequent genome-wide epigenetic studies based on Chinese populations.

## Results

**Figure 1:**
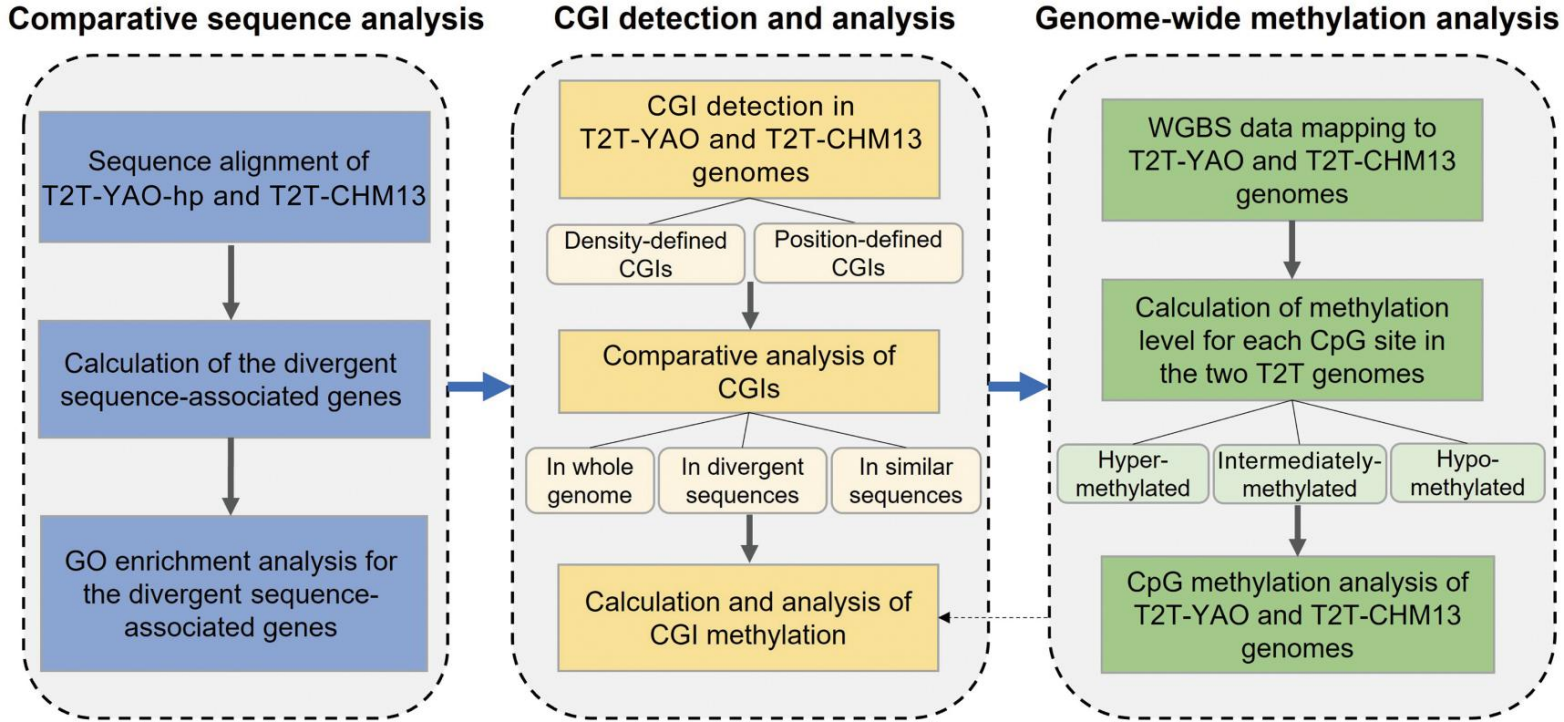
Workflow of the study GO, Gene Ontology; T2T, telomere-to-telomere; WGBS, whole-genome bisulfite sequencing; CGI, CpG island.

In order to address the three scientific questions, we analyzed the two genomic references, their CGI distribution, and genome-wide CGI-associated methylation (**Figure 1**).

### Comparative sequence analysis

To address our first question, we aligned the T2T-YAO-hp and T2T-CHM13 genomes and functionally categorized the divergently enriched genes from the T2T-YAO-hp genome (-hp stands for the haploid sequence from the offspring of the triad family T2T-YAO assemblies; T2T-YAO-hp and T2T-YAO used here are equivalent unless otherwise specified).

#### Sequence differences between T2T-YAO-hp and T2T-CHM13

**Figure 2:**
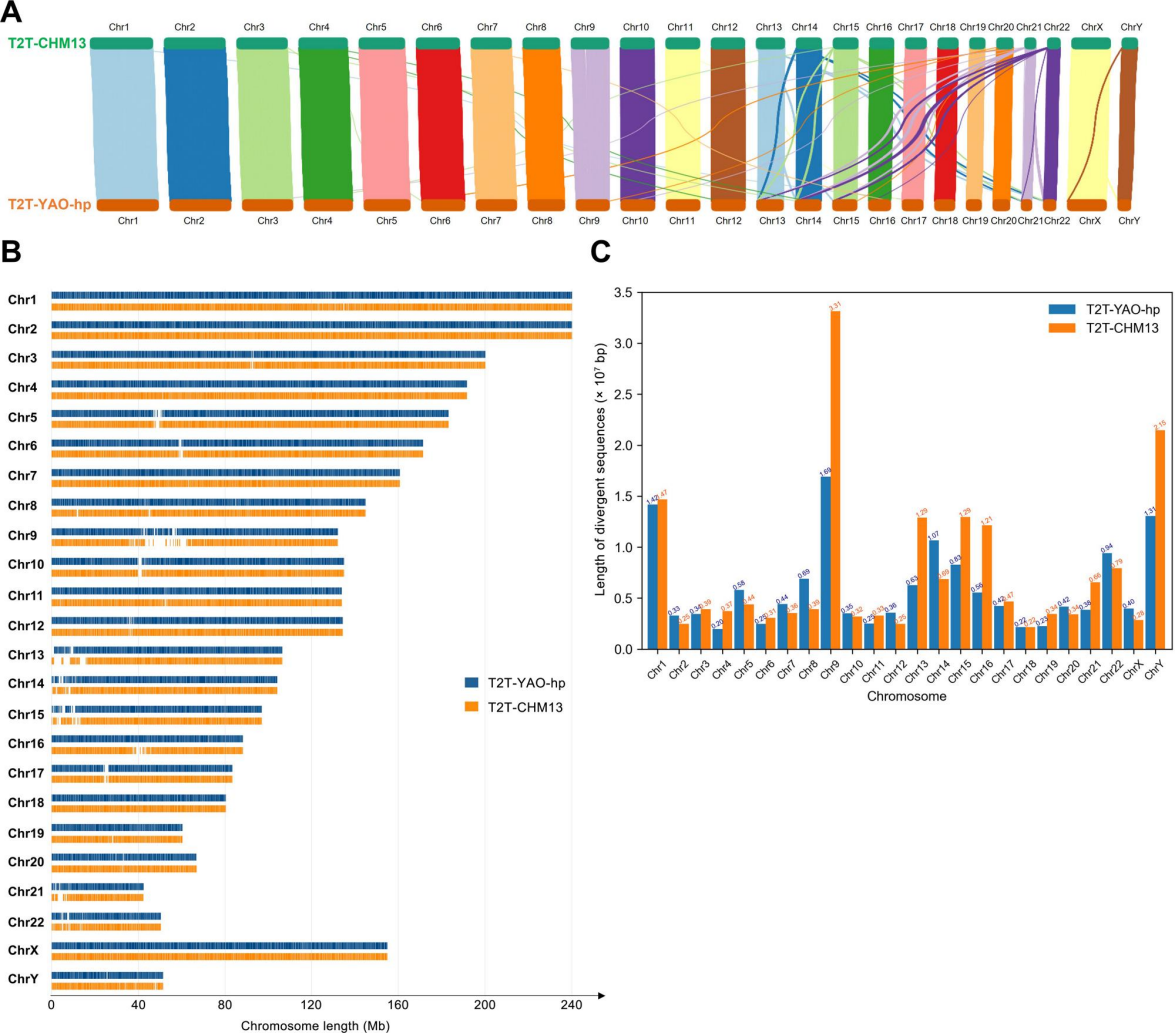
Sequence alignment between the T2T-CHM13 and T2T-YAO-hp genomes **A**. A collinearity map of the genome alignment. Colored lines link the high-identity or collinear sequence segments of the two compared genomes, with the line thickness as a similarity measure. **B**. Visualization of sequence comparison across chromosomes. Blue and orange highlight similar sequences as vertical bars of the T2T-CHM13 and T2T-YAO-hp genomes, respectively. Blank bars represent divergent sequences of the two genomes. **C**. Divergent sequence length statistics of the two genomes. Blue and orange bars represent the divergent sequence lengths of the T2T-CHM13 and T2T-YAO-hp genomes in different chromosomes, respectively. Chr, chromosome.

First, from the sequence alignment (the alignment results are detailed in Tables S1 and S2), as shown in **Table 1**, the T2T-CHM13 genome has approximately 54.6 Mb more sequences as compared to the T2T-YAO-hp genome. The uniquely divergent sequences account for 5.751% and 4.665% of the full-length T2T-CHM13 and T2T-YAO-hp genomes, respectively. Therefore, the difference between the two genomes is approximately 4.7%–5.8%. Second, using NGenomeSyn [23], we created a collinearity map of the two genomes and observed that the two sequences are very similar both in identity and chromosome length (**Figure 2**) in general [24]. The divergent sequences between the two genomes are mainly concentrated in specific regions of each chromosome (Figure  2B). For instance, some of the divergent sequences of chromosomes 15 and 22 are enriched at the their left telomeric ends, whereas others, such as chromosome 9, have those located near the centromeres. The examples of some longest diverge sequence segments include 3.31 × 10^7^ bp *vs*. 1.69 × 10^7^ bp of chromosome 9 and 2.15 × 10^7^ bp *vs.* 1.31 × 10^7^ bp of chromosome Y; both are longer in the T2T-CHM13 genome (Figure  2C).

**Table 1:** Sequence differences between the T2T-CHM13 and T2T-YAO-hp genomes

	Total length (bp)	Divergent sequence length (bp)	Divergent sequence proportion	Similar sequence length (bp)
T2T-CHM13	3,117,275,501	179,281,068	5.751%	2,937,994,433
T2T-YAO-hp	3,062,707,975	142,866,485	4.665%	2,919,841,490

*Note*: We use NUCmer [41] to compute similar sequences between the two reference genomes and call other remaining sequences as divergent sequences.

#### Divergent sequence-associated genes

**Figure 3:**
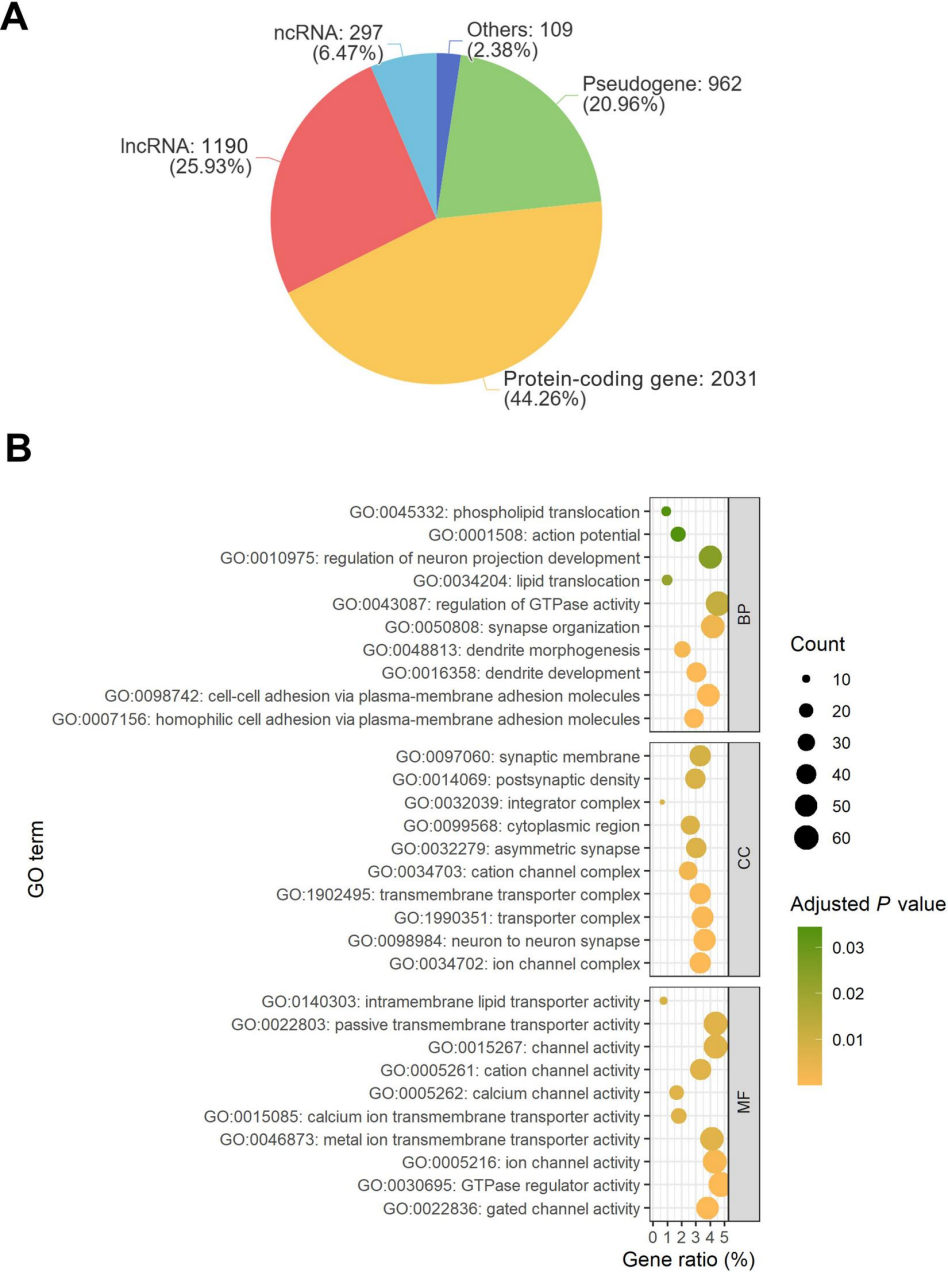
GO enrichment of divergent sequence-associated genes **A**. Proportion of the types of divergent sequence-associated genes in these two reference genomes. **B**. GO enrichment analysis of divergent sequence-associated protein-coding genes. Size and color of the circle represent the number of enriched genes and the statistical significance value (adjusted *P* value) [22,42] of the current GO term, respectively. ncRNA, non-coding RNA; lncRNA, long non-coding RNA; BP, biological process; MF, molecular function; CC, cellular component.

We categorized the divergent sequence-associated genes of the two genomes (**Figure 3**A; Table S3). The results show that most of the divergent sequence-associated genes are protein-coding genes (44.26%), followed by lncRNAs (25.93%). Our GO enrichment analysis for the protein-coding genes shows that most of the significantly enriched GO terms are associated with certain functions of the nervous system, such as “homophilic cell adhesion via plasma-membrane adhesion molecules”, “ion channel complex”, and “gated channel activity” in the biological process (BP), cellular component (CC), and molecular function (MF) categories [25], respectively (Figure  3B).

### CGI prediction and analysis

#### CGI prediction results

We employed the rule-based method [12,19] and distance-based clustering method [12,19] to predict the number of CGIs and the average CGI length in the T2T-YAO-hp and T2T-CHM13 genomes (**Table 2**, Table S4), and showed the distribution of CGIs on each of the chromosomes in the two genomes (Figure S1). For the density-defined CGIs, the total number and average length of CGIs predicted in the T2T-YAO-hp genome were both less than those predicted in the T2T-CHM13 genome (CGI count: 84,471 *vs.* 87,788; average length: 391 bp *vs.* 407 bp) (Table 2). For the position-defined CGIs, the total number of CGIs predicted in the T2T-YAO-hp genome was greater than that predicted in the T2T-CHM13 genome under the default parameter of “*d* = 50 bp” (222,421 *vs.* 220,736), but it was less in the T2T-YAO-hp genome under the parameter of “*d* = 25 bp” (161,997 *vs.* 174.594) or “*d* = 12 bp” (96,449 *vs.* 101,972); by contrast, the average lengths of CGIs predicted in the T2T-YAO-hp genome were collectively longer than those predicted in the T2T-CHM13 genome under different parameters (*d* = 50 bp: 261 bp *vs.* 258 bp; *d* = 25 bp: 60 bp *vs.* 58 bp; *d* = 12 bp: 24 bp *vs.* 23 bp) (Table 2). We also predicted all CGIs related to divergent sequences of the two genomes (Table S5).

**Table 2:** CGI statistics of the T2T-CHM13 and T2T-YAO-hp genomes

Category		Density-defined CGI	Position-defined CGI		
			*d* = 50 bp	*d* = 25 bp	*d* = 12 bp
T2T-CHM13	CGI count	87,788	220,736	174,594	101,972
	Average CGI length (bp)	407	258	58	23
T2T-YAO-hp	CGI count	84,471	222,421	161,997	96,449
	Average CGI length (bp)	391	261	60	24

*Note*: “*d*” denotes the maximum distance between neighboring CpGs in the position-defined CGIs. GGI, CpG island.

**Figure 4:**
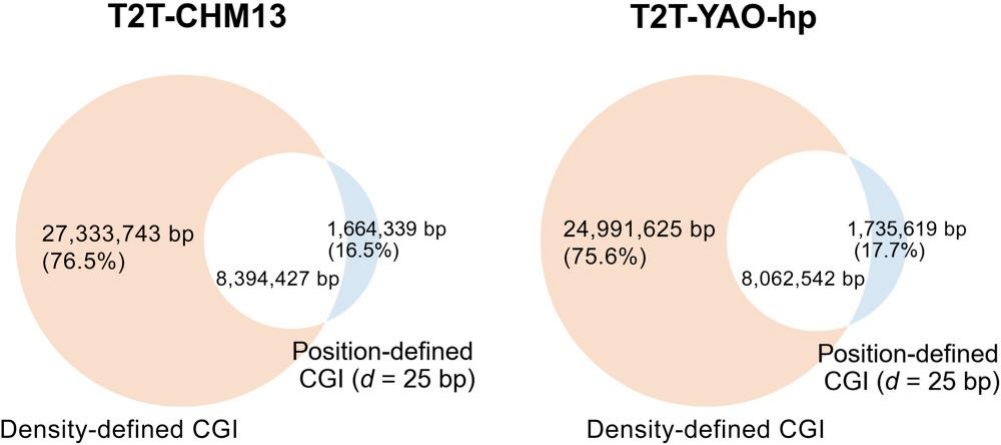
Overlap of sequences between the density-defined and position-defined CGIs The white part represents the length of sequences overlapped between position-defined (*d* = 25 bp) and density-defined CGIs. The orange and blue parts represent the length of sequences unique to the density-defined and position-defined (*d* = 25 bp) CGIs, respectively.

To study the relationship between position-defined CGIs and density-defined CGIs for the same genome, we investigated sequences that intersect the two groups (for the position-defined CGIs, *d* = 25 bp) for both genomes. As shown in **Figure 4**, the total length of density-defined CGIs is much longer, approximately three times that of position-defined CGIs for each genome [*e.g.*, (24,991,625+8,062,542)/(8,062,542+1,735,619)=3.37 for the T2T-YAO-hp genome]. In addition, although most of the position-defined CGIs overlap with the density-defined CGIs, there is still a fraction (16.5% for T2T-CHM13 and 17.7% for T2T-YAO-hp) that is not predicted by the rule-based CGI predicting method.

#### CGI feature comparison

**Figure 5:**
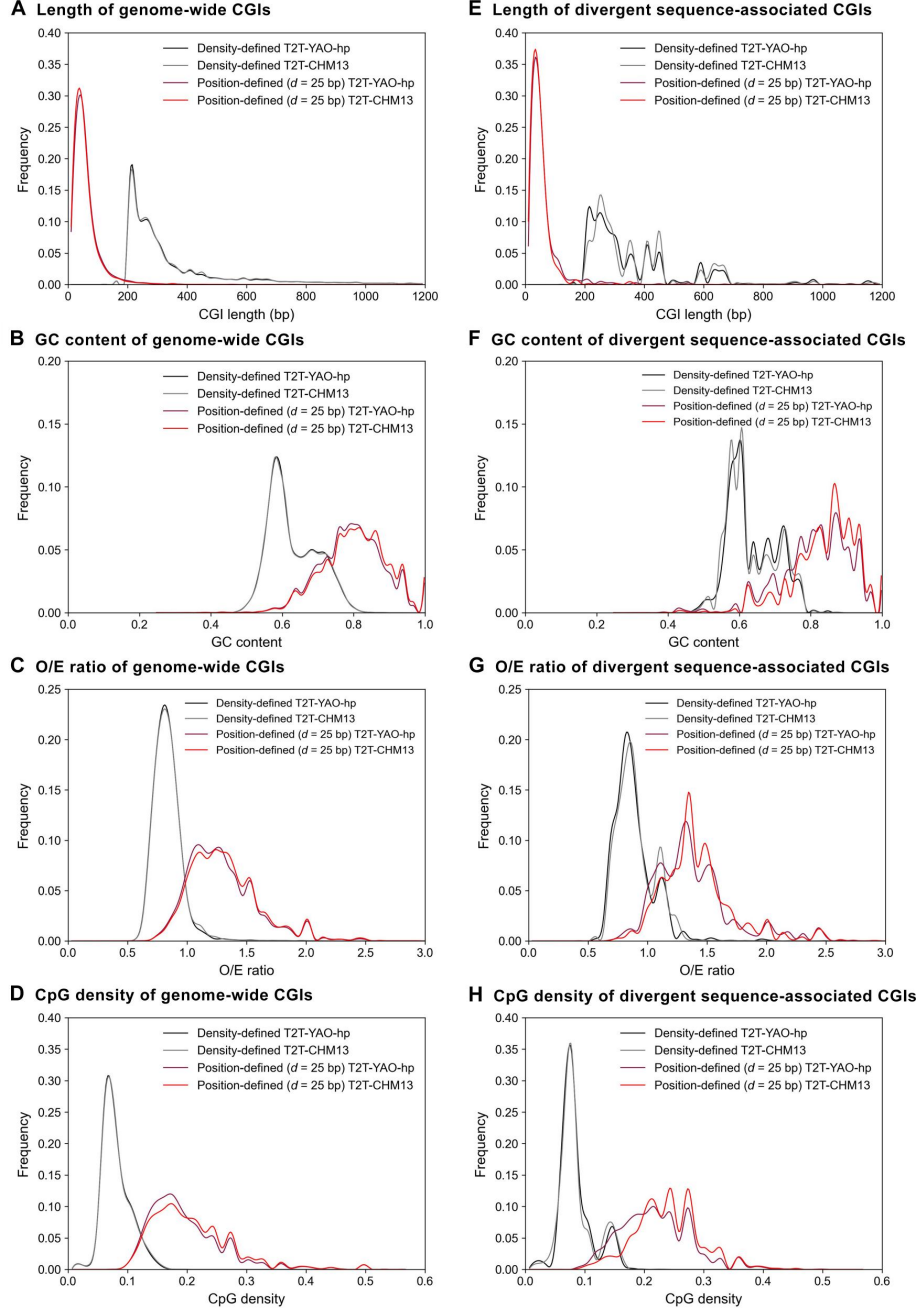
Comparison of CGI features between the T2T-CHM13 and T2T-YAO-hp genomes **A**. Length of genome-wide CGIs. **B**. GC content of genome-wide CGIs. **C**. O/E ratio of genome-wide CGIs. **D**. CpG density of genome-wide CGIs. **E**. Length of divergent sequence-associated CGIs. **F**. GC content of divergent sequence-associated CGIs. **G**. O/E ratio of divergent sequence-associated CGIs. **H**. CpG density of divergent sequence-associated CGIs. O/E, observed CpG/expected CpG.

To compare the differences in CGIs between the two genomes, we calculated four commonly used CGI features, *i.e.*, CGI length, GC content, CGI O/E ratio, and CpG density [2,26], and showed the feature distribution of genome-wide and divergent sequence-associated GCIs predicted by rule-based method or distance-based clustering method under parameters of “*d* = 12 bp”, “*d* = 25 bp”, and “*d* = 50 bp” (**Figure 5**, Figure S2). Except the grouping of density-defined or position-defined CGIs, there is no obvious difference in the distribution of the four commonly used CGI features between the two genomes for genome-wide CGIs (Figure  5A–D, Figure S2A–D). For example, in both genomes, the peak lengths of the density-defined CGIs and the position-defined CGIs (*d* = 25 bp) are approximately 210 bp and 30–50 bp, respectively (Figure  5A). However, there is a greater difference in the distribution of the four commonly used CGI features of the divergent sequence-associated CGIs between the two genomes than that in the genome-wide CGIs (Figure  5E–H, Figure S2E–H). For instance, the density-defined CGIs in the T2T-CHM13 genome have two peaks in high GC content regions (from 50% to 60%), whereas the density-defined CGIs in the T2T-YAO-hp genome have a single peak in the same regions. In addition, the position-defined CGIs in the divergent sequences, except for a small difference in the distribution of CGI length, have significantly different distributions for GC content, O/E ratio, and CpG density between the two genomes (Figure  5E–H, Figure S2E–H; Table S6). Moreover, for both genome-wide CGIs and divergent sequence-associated CGIs, the distance-based clustering method is more likely to identify CGIs with shorter length as well as higher GC content, O/E ratio, and CpG density than the rule-based CGI predicting method (Figure 5, Figure S2).

### Methylation analysis and comparison

To address our third question, we compared not only the number of C sites (Figure S3) and the methylation levels of all CpG sites, but also the methylation levels of genome-wide CGIs between the T2T-YAO-hp and T2T-CHM13 genomes.

#### CpG methylation levels

**Figure 6:**
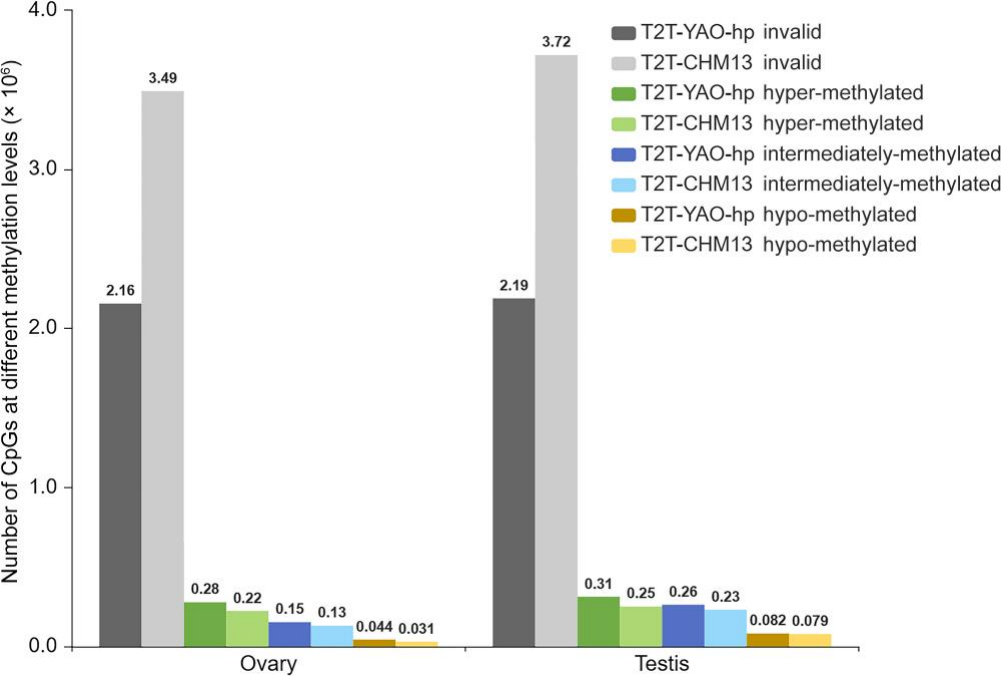
Comparison of CpG methylation levels of divergent sequences between the T2T-CHM13 and T2T-YAO-hp genomes WGBS data from ovary or testis are mapped to the divergent sequences in the T2T-YAO-hp and T2T-CHM13 genomes, respectively. CpG sites that fail to be mapped by the WGBS data are denoted as invalid. CpG sites that are successfully mapped by the WGBS data are classified into hyper-methylated, hypo-methylated, and intermediately-methylated based on Equation 7.

We first determined the CpG methylation profiles of the whole genomes and divergent sequences for the T2T-YAO-hp and T2T-CHM13 genomes, based on the WGBS data of two germline tissues, ovary and testis (Figure S4). Then, we classified the CpG sites predicted in the similar (Figure S5) and divergent (**Figure 6**) sequences between the T2T-YAO-hp and T2T-CHM13 genomes into four groups based on their methylation levels in the WGBS data of ovary and testis: invalid, hyper-methylated, hypo-methylated, and intermediately-methylated. The number of CpG sites in the divergent sequences that fail to be mapped by the same WGBS methylation data (*i.e.*, invalid) is much lower in the T2T-YAO-hp genome than in the T2T-CHM13 genome in both germline tissues. For example, the number of CpG sites in the divergent sequences of the ovary that fail to be mapped in the T2T-YAO-hp genome is 2.16 × 10^6^, whereas that in the T2T-CHM13 genome is 3.49 × 10^6^ (Figure 6). Inversely, the number of CpG sites in the divergent sequences that are successfully mapped by the same WGBS methylation data in the T2T-YAO-hp genome is greater than that in the T2T-CHM13 genome at the hyper-methylated, hypo-methylated, and intermediately-methylated levels. In terms of quantity, the most notable difference is what found in the hyper-methylated divergent sequences (2.8 × 10^5^*vs*. 2.2 × 10^5^ for ovary; 3.1 × 10^5^*vs.* 2.5 × 10^5^ for testis; Figure 6).

#### CGI methylation levels

**Figure 7:**
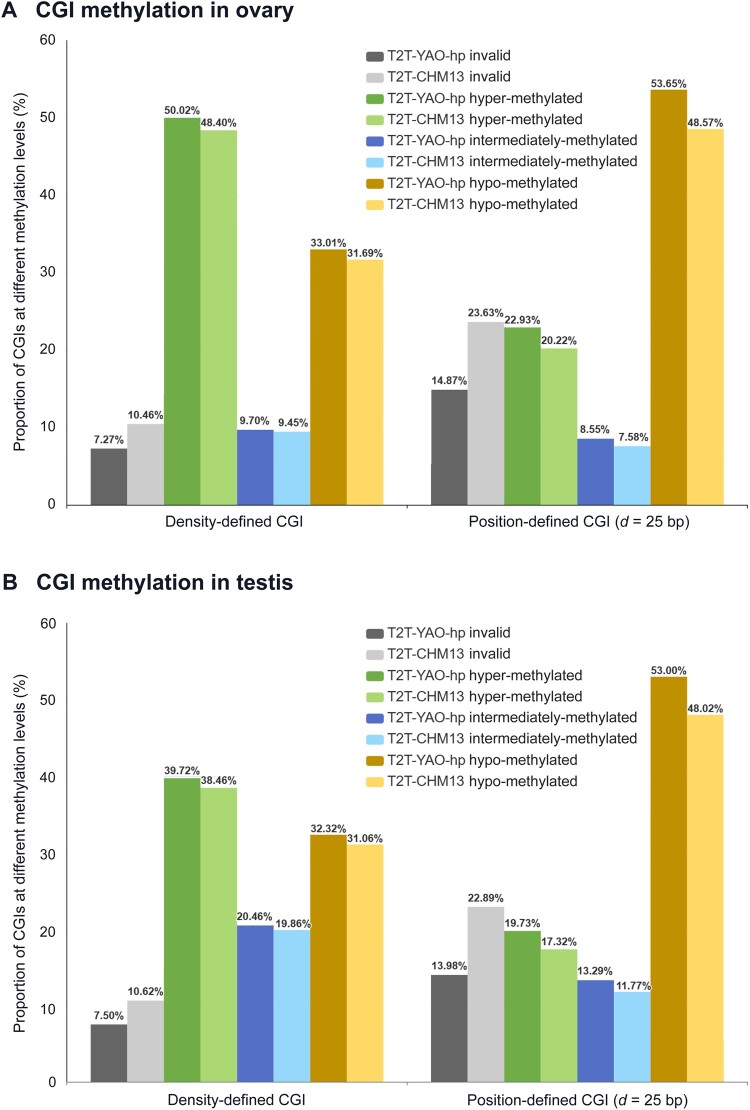
Comparison of genome-wide CGI methylation levels between the T2T-CHM13 and T2T-YAO-hp genomes **A**. CGI methylation levels annotated by the WGBS data from ovary. **B**. CGI methylation levels annotated by the WGBS data from testis. WGBS data from ovary or testis are mapped to the whole genomes of T2T-YAO-hp and T2T-CHM13. CGIs that fail to be mapped by the WGBS data are denoted as invalid. CGIs that are successfully mapped by the WGBS data are classified into hyper-methylated, hypo-methylated, and intermediately-methylated based on Equation 7.

We calculated the methylation levels of all density-defined and position-defined CGIs for the two reference genomes, and classified them into invalid, hyper-methylated, hypo-methylated, and intermediately-methylated groups as well (**Figure 7**, Figure S6). For both density-defined and position-defined CGIs, the proportions of CGI-invalid data (CGIs that fail to be mapped by WGBS data) are higher in the T2T-CHM13 genome than those in the T2T-YAO-hp genome: 10.46% *vs*. 7.27% for the density-defined CGIs and 23.63% *vs.* 14.87% for the position-defined CGIs in the ovary (Figure  7A); 10.62% *vs.* 7.50% for the density-defined CGIs and 22.89% *vs.* 13.98% for the position-defined CGIs in the testis (Figure 7B). By contrast, the proportions of hyper-methylated, intermediately-methylated, and hypo-methylated CGIs, predicted by both definitions, are slightly higher in the T2T-YAO-hp genome than those in the T2T-CHM13 genome, *e.g.*, 19.73% *vs.* 17.32%, 13.29% *vs.* 11.77%, and 53.00% *vs.* 48.02% for the hyper-methylated, intermediately-methylated, and hypo-methylated position-defined CGIs between the two genomes in the testis, respectively (Figure  7B). Notably, the density-defined CGIs appear to have the highest proportion of hyper-methylation for both genomes, whereas the position-defined CGIs appear to have the highest proportion of hypo-methylation (Figure 7).

## Discussion and conclusion

The aim of this study is to emphasize merits and specific applications of a population-centric reference genome by comparing the sequence divergence, as well as various epigenetic parameters, between the T2T-YAO and T2T-CHM genomes. We further provide the evidence suggesting the necessity of using T2T-YAO as a reference genome for future genome-wide genetic and epigenetic studies in Chinese populations. We start with the two high-quality genome sequences to compute the genome-wide C and CpG sites and further categorize them into two groups of CGIs, position-defined and density-defined. After the three-round stratification, we present the divergent sequence-associated genes and conclude that these genes are mostly neurological in function. In addition, we map the methylation levels of CpGs and CGIs for the two reference genomes using selected public WGBS data.

The overall sequence divergence between the two reference genomes is significant and interesting, albeit only 4.7%–5.8% (minimal alignment length of 5000 bp and identity cutoff of 90%). Such a loosely-defined difference, generally speaking, is mostly population-associated variations between the northern Chinese and European populations [9,10]. Future studies will have to expand empirical data into within-population variations based on big data for their detailed categorization of functional relevance. The current divergent sequences appear to non-uniformly spread out into certain chromosomal regions within a few chromosomes and their specific regions, such as the cases involving chromosomes 9 and Y; such a distribution also suggests population-associated selection and recombination. Furthermore, the GO enrichment of divergent-sequence-associated genes specific to the T2T-YAO genome indicates a strong preference for specific molecular mechanisms related to neuronal cells and synapses, providing clues for future in-depth investigation.

Our genome-wide CGI study between the two genomes indicates that two-fold CGI stratifications are necessary for precision methylation mapping. It is important to start methylation mapping from all potential methylation sites that are ensured only by T2T population-specific genomes. The two CGI definitions and their grouping schemes, such as the distance (12 bp, 25 bp, and 50 bp) and the density (high, moderate, and low), provide matrices for pattern and rule recognitions, which are used to identify genes and other genomic elements and features for further functional scrutinization. In addition, after the application of the two CGI definitions, there is still a significant fraction of the C and CpG sites to be further annotated, leveraging on the complete genomes, as some of these sites are actually methylated.

From the methylation mapping of the two datasets representing the two germline tissues, we have learnt several lessons. First, we have expected more CpG sites to be mapped in the T2T-CHM13 reference from the methylation data of the European origins, since its DNA is from a single homozygous complete hydatidiform mole (46, XX) and the Y sequence is from another assembly of similar origin. However, to our surprise, the number of CpG methylation sites mapped to the divergent sequences in the T2T-YAO genome is greater than that in the T2T-CHM13 reference. Second, the CpG sites that could not be mapped using the gender-specific methylation data also exhibit a directional bias, with a higher mapping success in the T2T-YAO reference. These subjective mapping results lead to an across-the-board conclusion that methylation patterns are reference sensitive regardless the methylation levels. Third, we also show apparent differences of CGI methylation between the two CGI definitions, and the rate varies among different methylation levels.

After all, there are still several questions that need further exploration. First, the success of large-scale genome-wide epigenomic studies relies on high-quality and population-specific reference genomes, and there are more much more references to be constructed for human methylation atlas of different populations. Second, novel and more thorough CpG and CGI definitions are needed for understanding the relatedness of data to be mapped and their references. Since artificial intelligence (AI) has been gradually used in genome-wide methylation-associated studies [27,28], determining how to use AI techniques [29–32] such as neural networks to investigate the relationship between sequence features, CGI, methylation, and expression specificity of the human genome has become a future research direction. Finally, genome-wide CGI prediction and methylation analysis are not only data-intensive but also time-consuming. However, there is currently no such web service that collects and manages CGI and methylation annotation data for the Chinese T2T genome. Therefore, we will build a web service for online computation, data sharing, and visualization based on high-performance computing [33,34], such as GPU acceleration and distributed computing, to provide a data foundation and an online platform for further study.

## Materials and methods

### Data source

The human GRCh38 and T2T-CHM13 (v2.0) genome sequences were downloaded from the National Center for Biotechnology Information (NCBI) databases [35], and the Chinese T2T-YAO-hp genome sequence was downloaded from the Genome Warehouse database (GWH: GWHDQZI00000000) [36]. The CGI data of the T2T-CHM13 genome, which were predicted based on the Gardiner-Garden and Frommer (GGF) standard [37] in University of California Santa Cruz (UCSC) Genome Browser database [38], were used in this study. The WGBS data of the ovary and testis tissues were downloaded from the Encyclopedia of DNA Elements database (ENCODE: ENCSR417YFD for ovary from a European donor; ENCSR806NNG for testis from an American individual) [39].

### Comparative sequence analysis

#### Statistics of all C sites

We counted and compared the number and distribution of C sites that can be methylated among the T2T-YAO-hp, T2T-CHM13, and GRCh38 genomes. Since most DNA methylation sites found in mammals involve CpG dinucleotides [40], we categorized C sites into three different contexts: CpG, CHG, and CHH, where H represents A, C, or T.

#### Genome alignment and analysis

We used NUCmer, a sequence alignment algorithm of the MUMmer system [41], to carry out genome-wide alignment and analysis for the T2T-YAO-hp and T2T-CHM13 genomes (Figure S7). The key parameters for NUCmer to calculate similar and divergent sequences were set as follows: minimum alignment length = 5000 bp, minimum alignment similarity = 90%. The similar sequences are those regions identified by NUCmer as aligning between the two genomes. Conversely, divergent sequences are those regions that do not meet the aforementioned alignment criteria, thus representing unique sequences present in each genome. The GO enrichment analysis of the divergent sequence-associated genes was performed using the clusterProfiler algorithm [42].

### CGI definition and analysis

The CGIs predicted by the classical rule-based method and the distance-based clustering method are denoted as density-defined and position-defined CGIs, respectively, as previously described [26,37]. Additionally, the distance-based clustering method is able to localize more short CGIs with potential gene regulatory functions or closely correlated with gene expression specificity in the human genome [2,11,12]. To comprehensively compare CGI features between the T2T-YAO-hp and T2T-CHM13 genomes, the density-defined and position-defined CGIs were predicted in both the whole genomes and the divergent sequences (Figure S8).

#### Density-defined CGI

Based on the GGF standard of CGIs [37], the sequence segments are analyzed by the CpGPlot algorithm [43]. The sequences that meet Equations 1–3 are denoted as density-defined CGIs.

#(1) $$\begin{array}{c} \mathrm{Length}\;\geq \;200\;\mathrm{bp} \end{array}$$

#(2) GC content ≥ 50%

#(3) $$\begin{array}{c} O/E\;\mathrm{ratio}=\frac{\mathrm{Number}\;\mathrm{of}\;\mathrm{CpG}}{\mathrm{Number}\;\mathrm{of}\;C\;\times \;\;\mathrm{Number}\;\mathrm{of}\;G}\;\times \;\mathrm{Length}\;\geq \;0.6 \end{array}$$

where Length is the nucleotide number of the analyzed gene sequence region, GC content is the proportion of G+C in the analyzed gene sequence region, and O/E ratio represents the ratio of observed CpGs to expected CpGs.

#### Position-defined CGI

The CpG clusters in the genome are first predicted by Equation 4, and then the predicted CpG clusters with small *P* values [26] (Equation 5) are considered as position-defined CGIs.

#(4) di=xi + 1-xi-1

#(5) P(d)=(1-p)d−1p

where x represents the coordinate of CpG, i represents the index of CpG, *P*(*d*) represents the probability of finding a distance *d* between neighboring CpGs, and *p* corresponds to the probability of CpGs in the sequence. Considering our previous findings that lineage-associated underrepresented permutations (LAUPs) are strongly associated with CGIs and the length of the shortest LAUPs of multiple species ranges from 10 bp to 14 bp, along with 25 bp serving as the statistical proportionality demarcation point for all CpGs and core-promoter-associated CpG regions in the human genome under the default setting of 50 bp in the CpGcluster algorithm [2,19], we predicted CGIs with *d* = 50 bp, *d* = 25 bp, and *d* = 12 bp (the average of 10 bp and 14 bp).

### Genome-wide methylation analysis

#### WGBS data mapping

The WGBS data from the ovary and testis were analyzed by gemBS [44], a large-scale WGBS data analysis method, to obtain the genome-wide and divergent-sequence methylation profiles by setting T2T-YAO-hp and T2T-CHM13 as reference genomes. The details of the procedure are described in Figure S9.

#### CGI methylation ratio computing

After obtaining the CGI prediction for the reference genome and the methylation ratio for each CpG site, the methylation ratio for each CGI is calculated as follows:

#(6) $$\begin{array}{c} \mathrm{mean}\_\mathrm{methylation}(\mathrm{cgi})=\frac{\sum_{\mathrm{cpg}\in \mathrm{cgi}}\mathrm{reads}\times \mathrm{methylation}\_\mathrm{ratio}(\mathrm{cpg})}{\sum_{\mathrm{cpg}\in \mathrm{cgi}}\mathrm{reads}} \end{array}$$

where *reads* represent the sequencing depth of WGBS for each CpG site in the CGI, and methylation_ratio(*cpg*) represents the methylation ratio of each CpG site derived from the gemBS parallelization algorithm.

#### Methylation level classification

According to the previous studies [2,45], the methylation ratio for each CpG site or CGI is classified into four methylation levels: invalid, hyper-methylated, hypo-methylated, and intermediately-methylated as below:

#(7) $$\text{Methylation level(}\text{chr},\text{p})=\left\{ \begin{array}{c} -1,\;\;\text{invalid (no reads mapped to this}\text{ cpg}\text{ or }\text{cgi})\\ {1,\;\;\text{hyper-methylated (methylation}}_{\text{ratio}\left(\text{chr},p\right)}>0.75)\\ {\;\;\;\;\;2,\;\text{hypo-methylated (methylation}}_{\text{ratio}\left(\text{chr},p\right)}<0.1)\;\;\\ 3,\;\text{intermediately-methylated (otherwise)} \end{array}\right.$$

where *chr* and *p* represent the chromosome and the coordinate of the CpG site, respectively.

## CRediT author statement

**Ming Xiao:** Conceptualization, Methodology, Software, Validation, Investigation, Data curation, Writing – original draft, Writing – review & editing, Visualization, Supervision, Project administration, Funding acquisition. **Rui Wei:** Conceptualization, Methodology, Software, Validation, Investigation, Data curation, Writing – original draft, Writing – review & editing, Visualization, Project administration. **Jun Yu:** Conceptualization, Writing – review & editing, Visualization, Supervision, Project administration. **Chujie Gao:** Formal analysis, Investigation, Resources, Data curation. **Fengyi Yang:** Formal analysis, Investigation, Resources, Data curation. **Le Zhang:** Conceptualization, Writing – review & editing, Visualization, Supervision, Project administration, Funding acquisition. All authors have read and approved the final manuscript.

## Supplementary material

Supplementary material is available at *Genomics, Proteomics & Bioinformatics* online (https://doi.org/10.1093/gpbjnl/qzae009).

## Competing interests

The authors have declared no competing interests.

## Supplementary Material

qzae009_Supplementary_Data
