# Supplementary figures and images for: CpG Island Definition and Methylation Mapping of the T2T-YAO Genome
Source: Genomics Proteomics Bioinformatics. 2024 Feb 1;22(2):qzae009. doi: 10.1093/gpbjnl/qzae009 (PMC12016031; doi:10.1093/gpbjnl/qzae009)

**A**

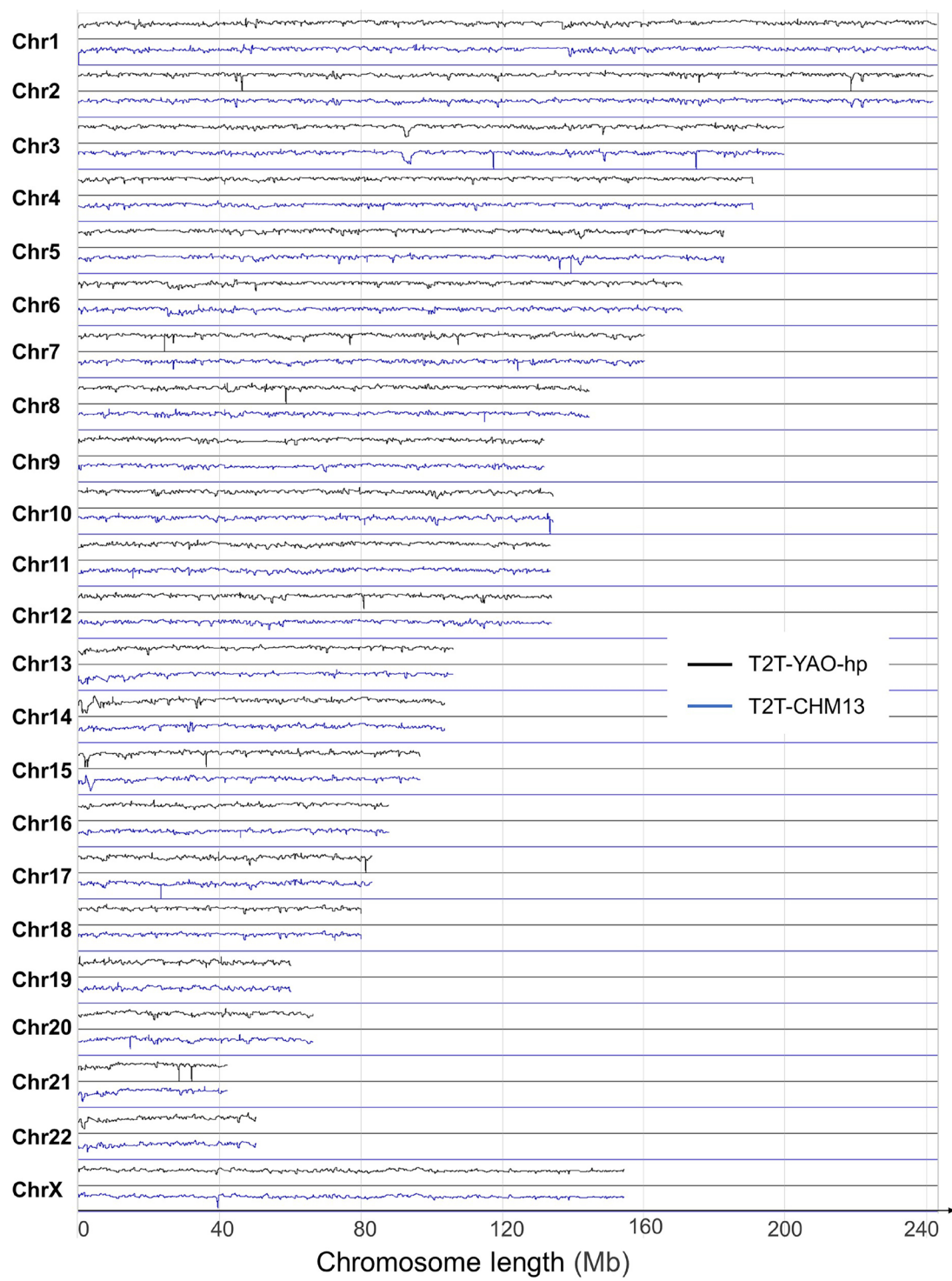

**B**

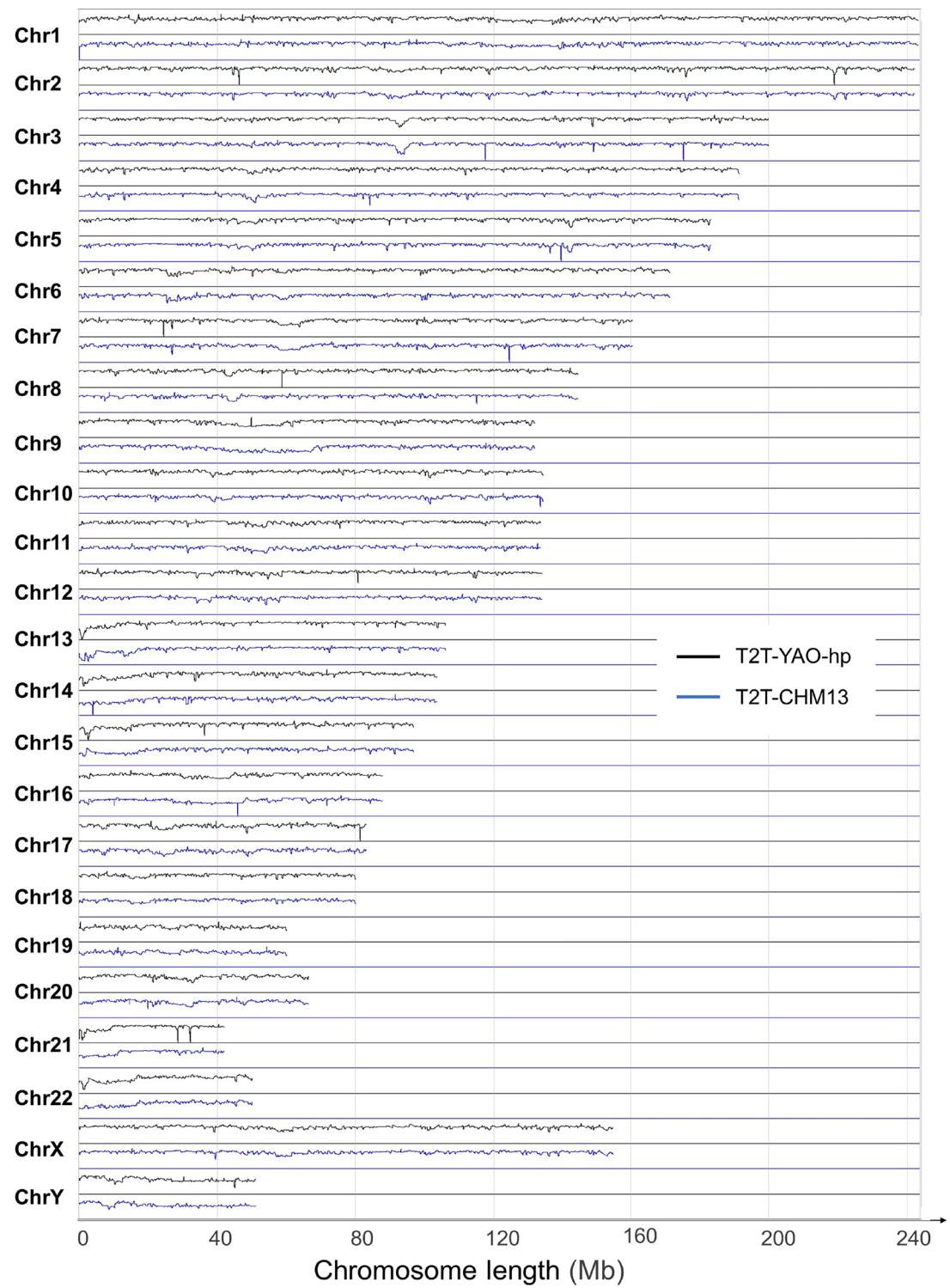

C

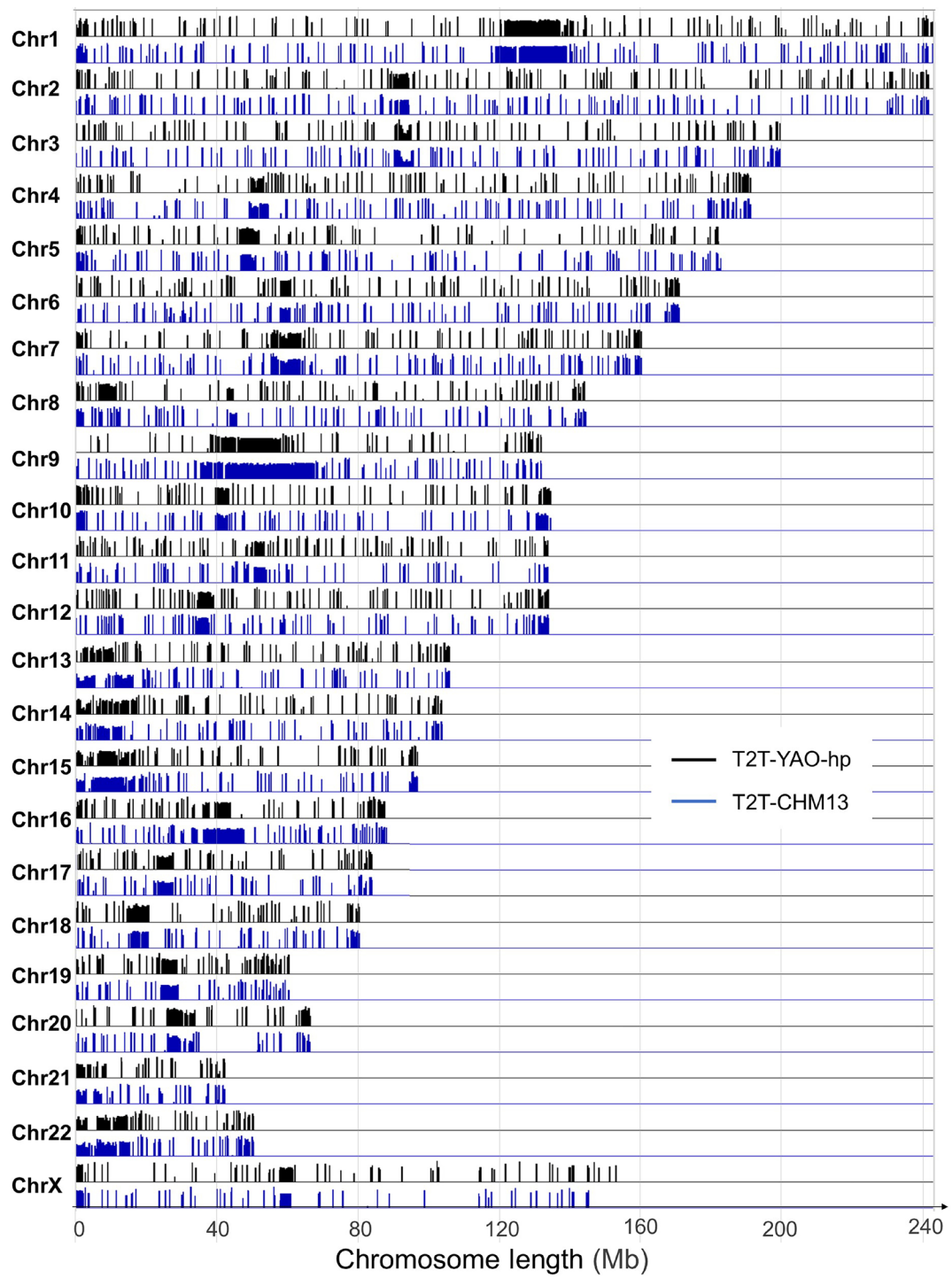

D

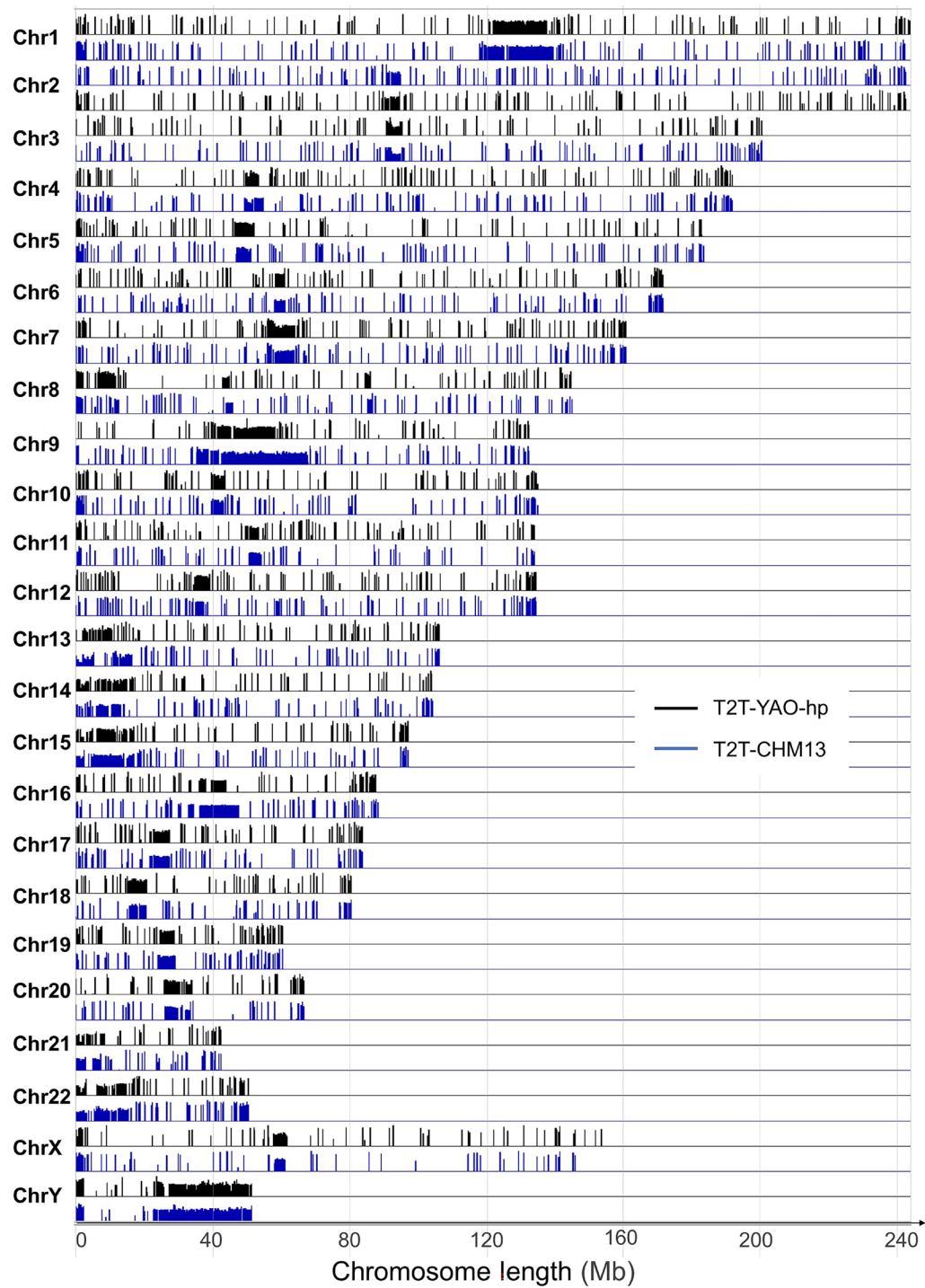

Supplement: qzae009_Supplementary_Data [file qzae009_supplementary_data.zip › Figure S4.pdf]

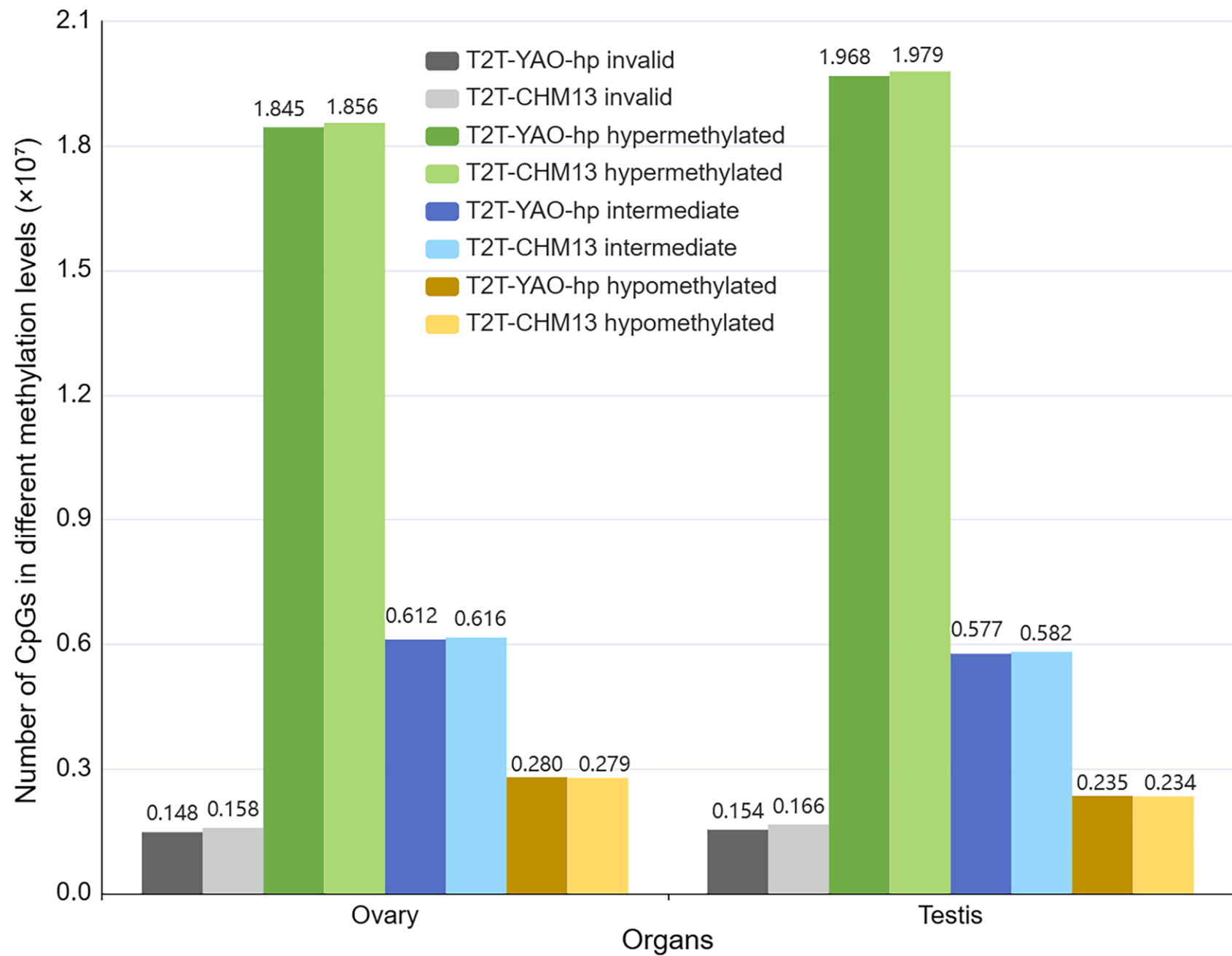

Supplement: qzae009_Supplementary_Data [file qzae009_supplementary_data.zip › Figure S5.pdf]

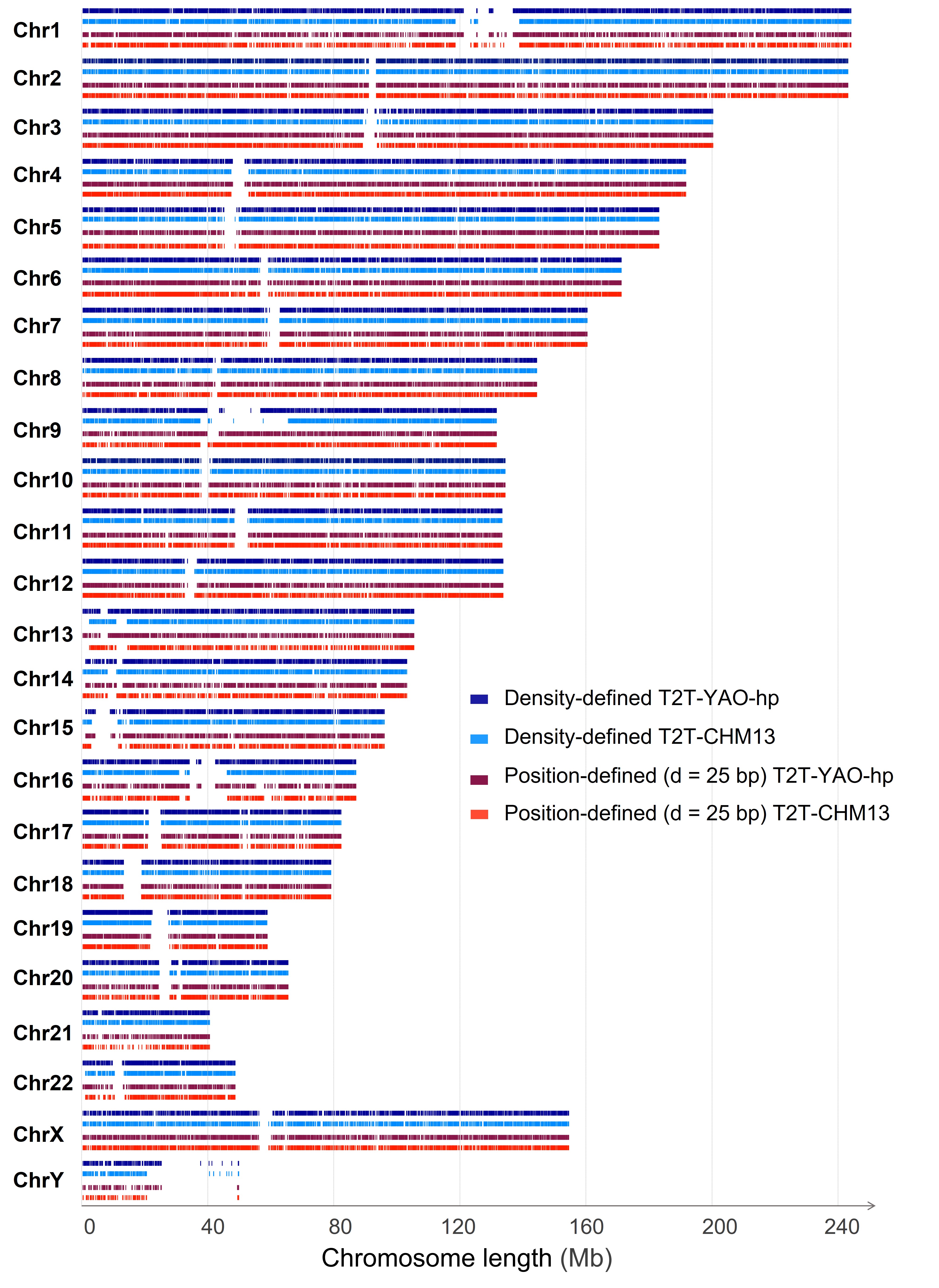

Supplement: qzae009_Supplementary_Data [file qzae009_supplementary_data.zip › Supplementary Figure S1.jpeg]

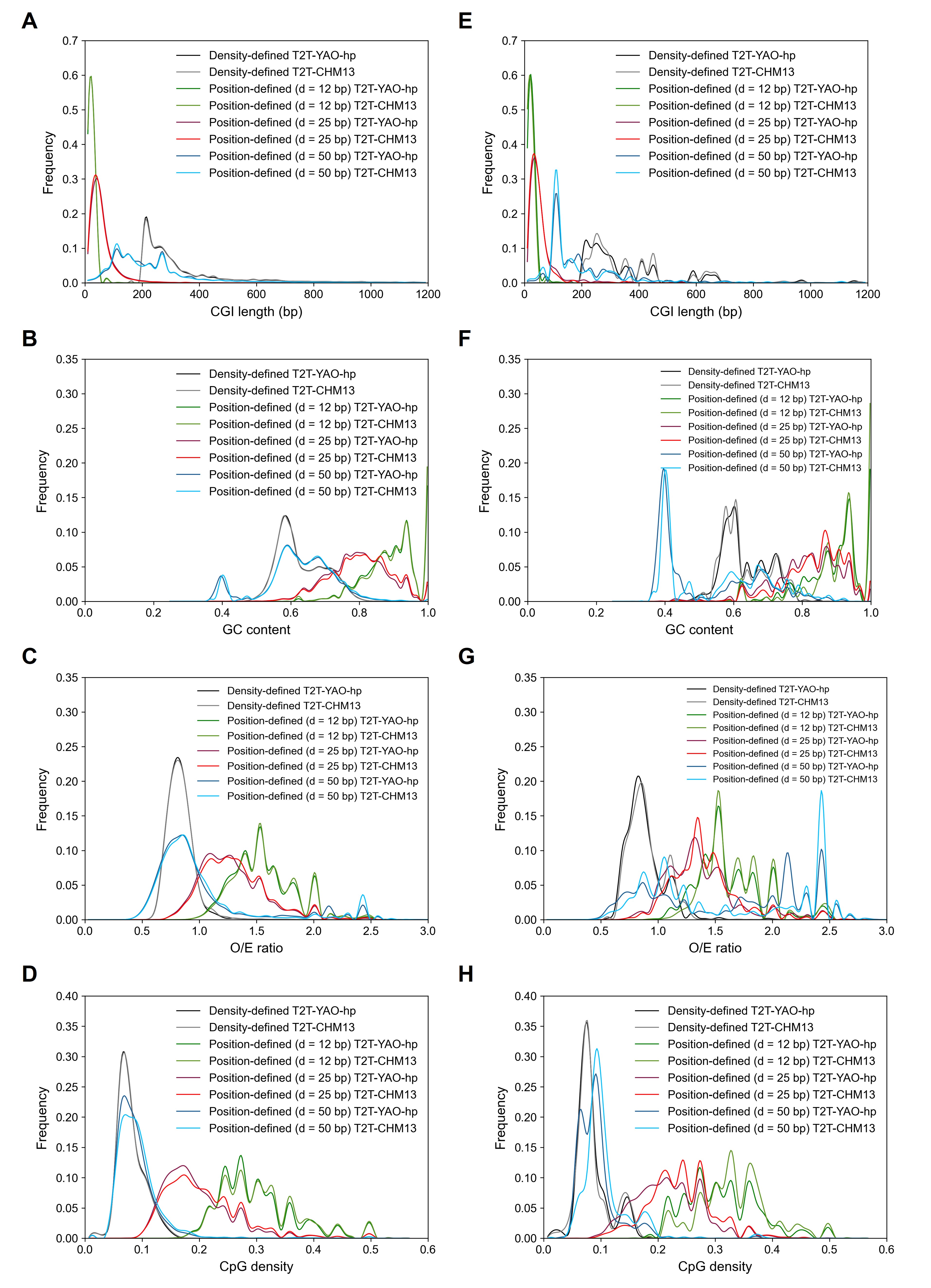

Supplement: qzae009_Supplementary_Data [file qzae009_supplementary_data.zip › Supplementary Figure S2.jpeg]

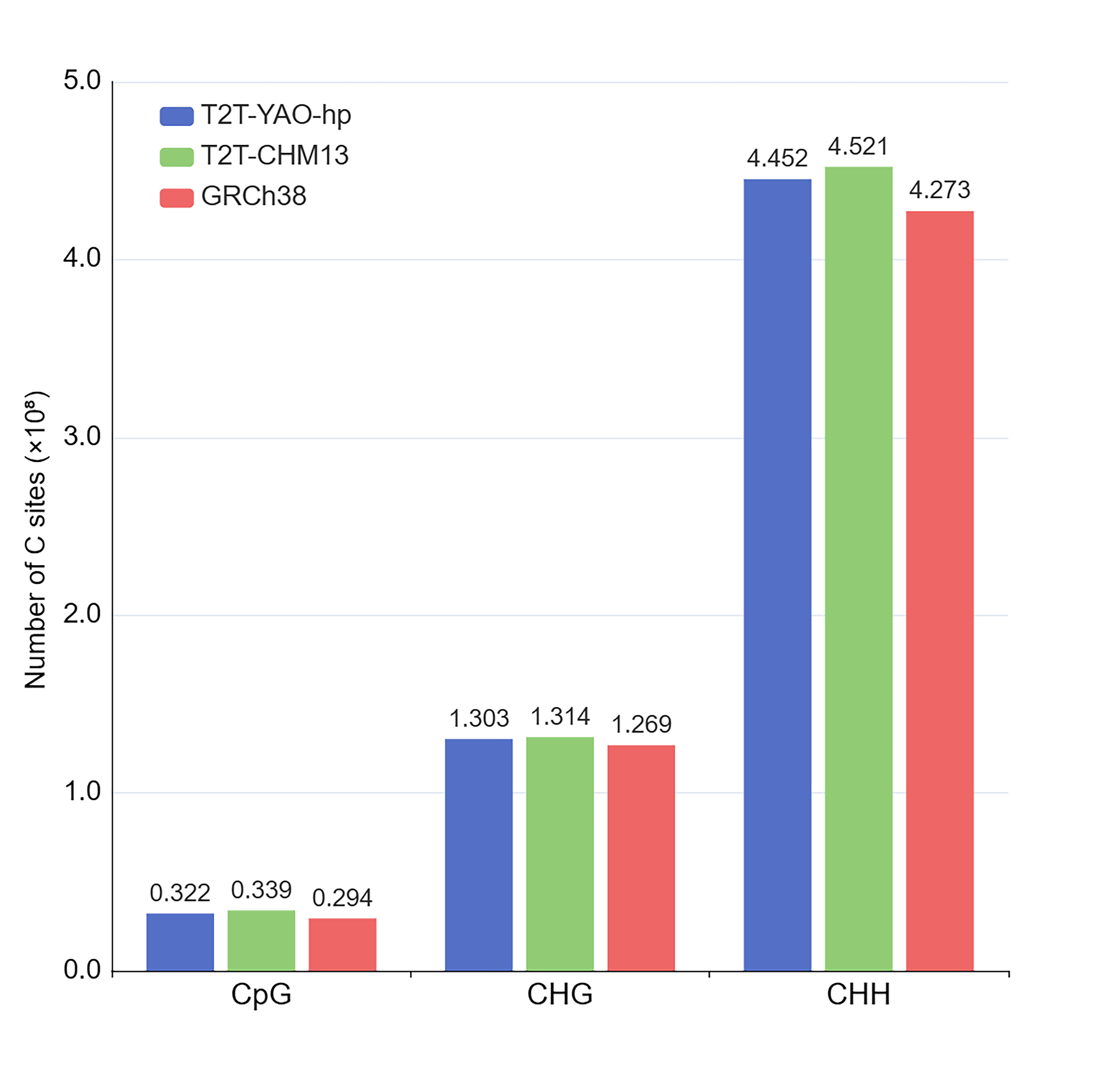

Supplement: qzae009_Supplementary_Data [file qzae009_supplementary_data.zip › Supplementary Figure S3.jpeg]

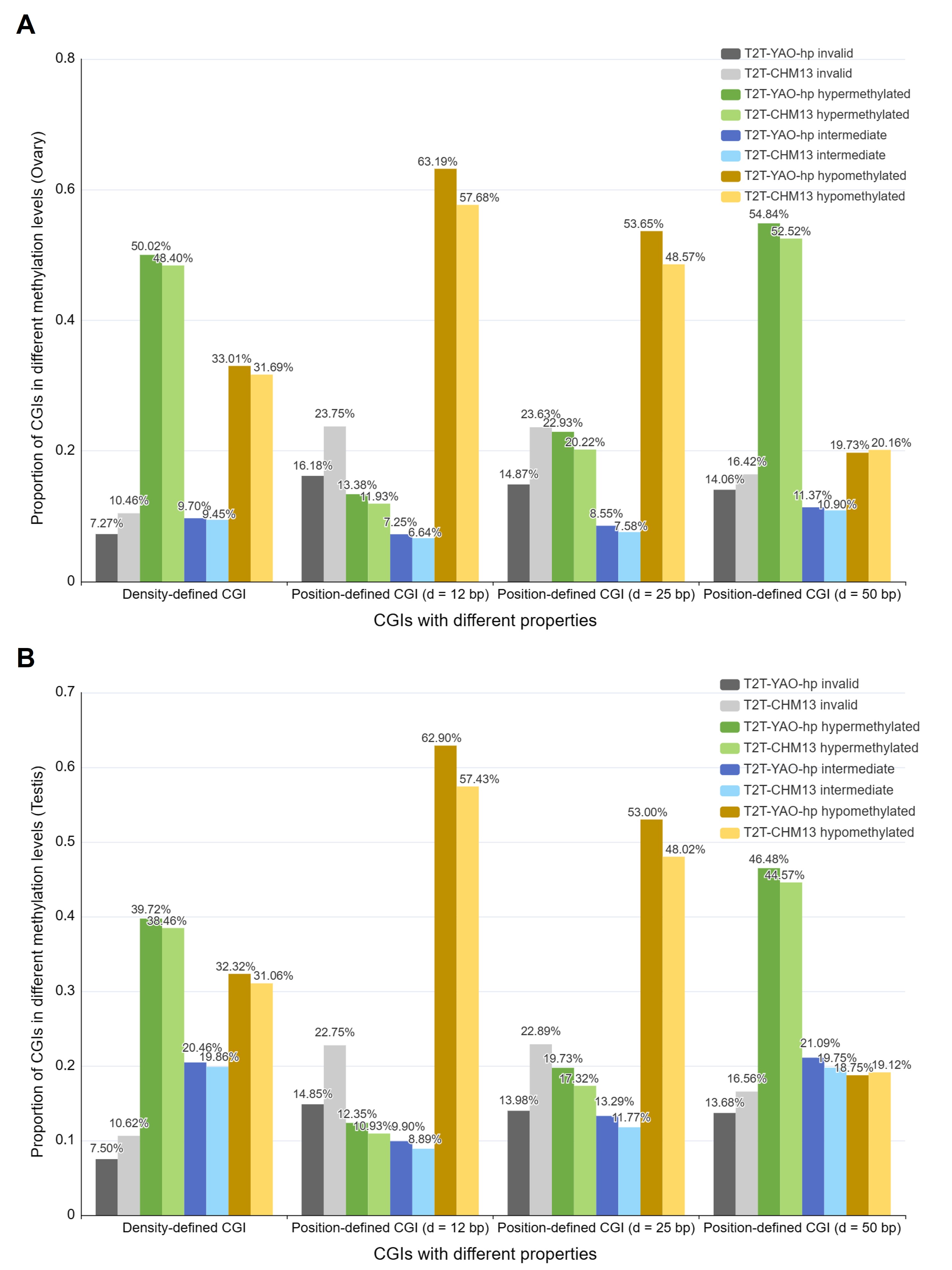

Supplement: qzae009_Supplementary_Data [file qzae009_supplementary_data.zip › Supplementary Figure S6.jpeg]

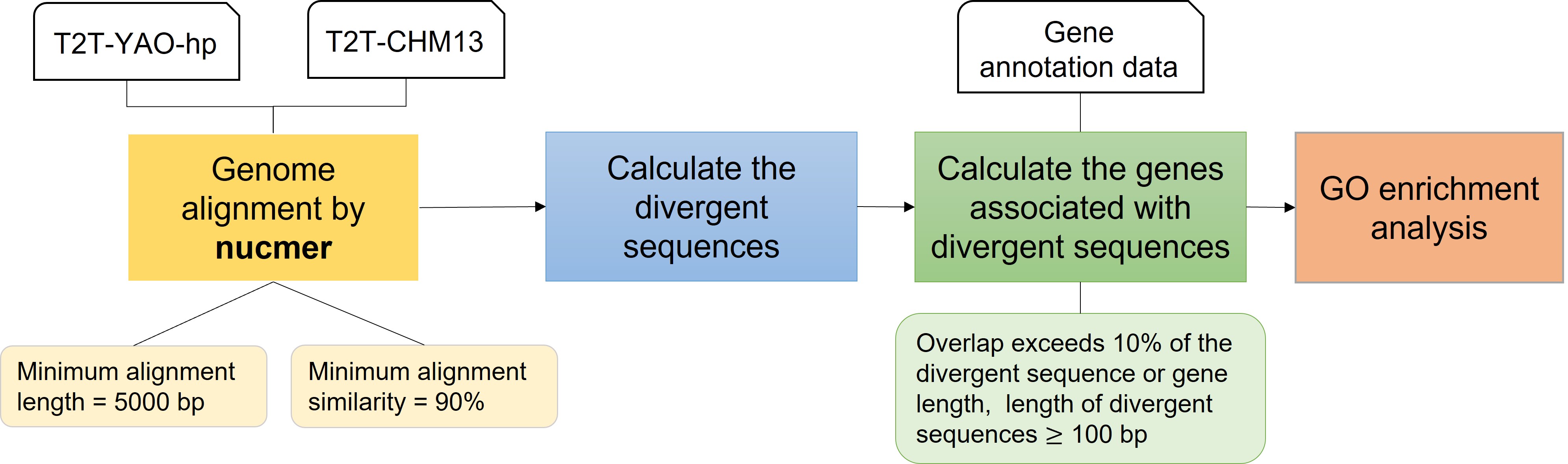

Supplement: qzae009_Supplementary_Data [file qzae009_supplementary_data.zip › Supplementary Figure S7.jpeg]

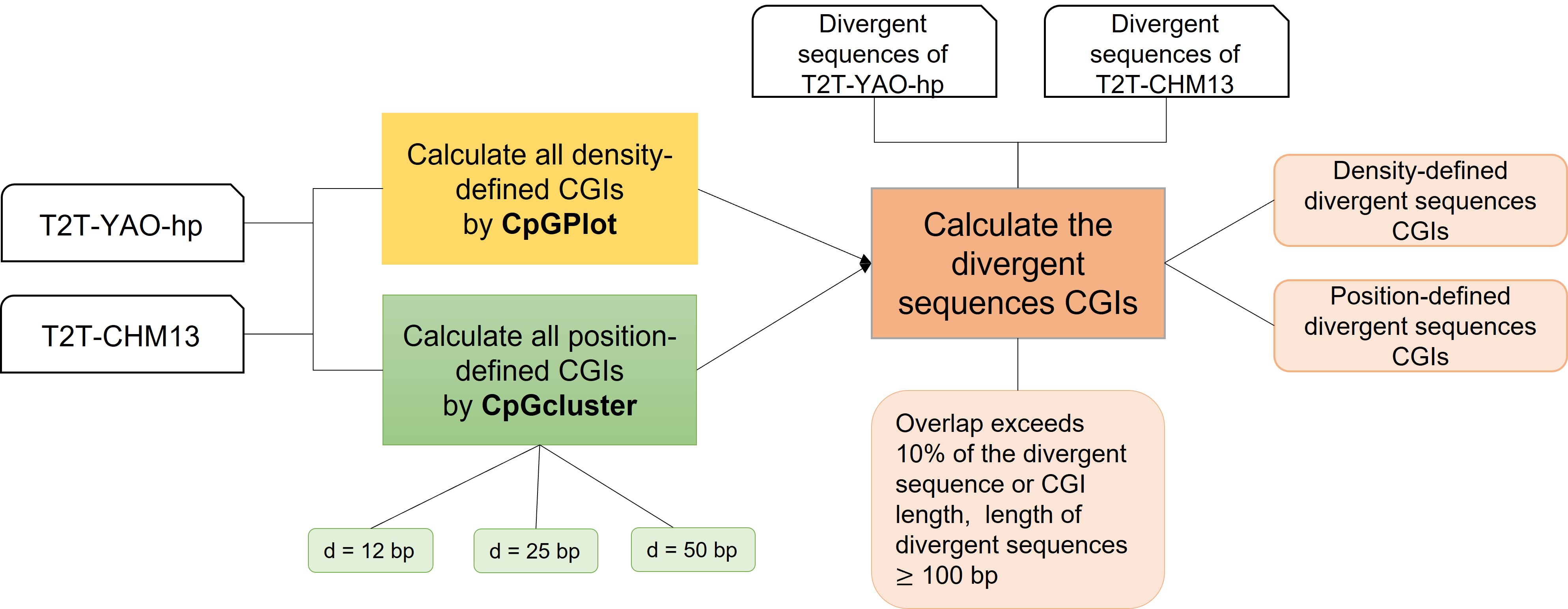

Supplement: qzae009_Supplementary_Data [file qzae009_supplementary_data.zip › Supplementary Figure S8.jpeg]

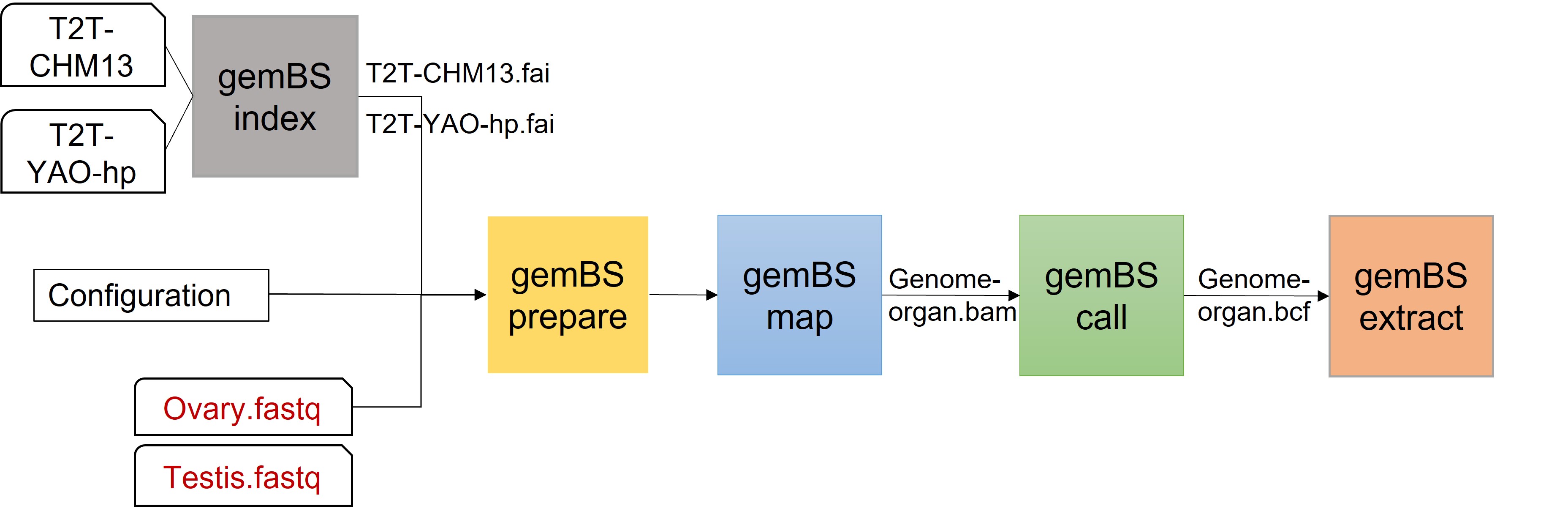

Supplement: qzae009_Supplementary_Data [file qzae009_supplementary_data.zip › Supplementary Figure S9.jpeg]
